# Estimating the individualized HIV-1 genetic barrier to resistance using a nelfinavir fitness landscape

**DOI:** 10.1186/1471-2105-11-409

**Published:** 2010-08-03

**Authors:** Kristof Theys, Koen Deforche, Gertjan Beheydt, Yves Moreau, Kristel van Laethem, Philippe Lemey, Ricardo J Camacho, Soo-Yon Rhee, Robert W Shafer, Eric Van Wijngaerden, Anne-Mieke Vandamme

**Affiliations:** 1 Rega Institute for Medical Research, Katholieke Universiteit Leuven, Leuven, Belgium; 2 ESAT, Katholieke Universiteit Leuven, Leuven, Belgium; 3 Instituto de Higiene e Medicina Tropical, Universidade Nova de Lisboa, Lisbon, Portugal; 4 Internal Medicine, University Hospitals Leuven, Leuven, Belgium; 5 Division of Infectious Diseases, Department of Medicine, Stanford University, Stanford, CA, USA

## Abstract

**Background:**

Failure on Highly Active Anti-Retroviral Treatment is often accompanied with development of antiviral resistance to one or more drugs included in the treatment. In general, the virus is more likely to develop resistance to drugs with a lower genetic barrier. Previously, we developed a method to reverse engineer, from clinical sequence data, a fitness landscape experienced by HIV-1 under nelfinavir (NFV) treatment. By simulation of evolution over this landscape, the individualized genetic barrier to NFV resistance may be estimated for an isolate.

**Results:**

We investigated the association of estimated genetic barrier with risk of development of NFV resistance at virological failure, in 201 patients that were predicted fully susceptible to NFV at baseline, and found that a higher estimated genetic barrier was indeed associated with lower odds for development of resistance at failure (OR 0.62 (0.45 - 0.94), per additional mutation needed, p = .02).

**Conclusions:**

Thus, variation in individualized genetic barrier to NFV resistance may impact effective treatment options available after treatment failure. If similar results apply for other drugs, then estimated genetic barrier may be a new clinical tool for choice of treatment regimen, which allows consideration of available treatment options after virological failure.

## Background

Management of antiviral resistance is an important consideration in the treatment of HIV-1 patients with antiviral drugs [1]. Facing high viral loads and fast replication rates, a combination of multiple drugs is needed to suppress viral replication so that the viral load in the plasma becomes undetectable. HIV-1 has a high mutation rate, and in conjunction with the large intra-host population and fast generation time [2], the virus is able to develop resistance mutations quickly. Therefore, a strict adherence to the treatment is regarded as crucial in the prevention of suboptimal drug concentrations and subsequent viral replication.

As part of the management of antiviral treatment, genotypic resistance testing is recommended when starting or switching treatment [3]. When virological failure is detected timely and a genotypic resistance test performed immediately, in many cases the test shows that the virus has developed resistance but not to all drugs in the regimen [4]. Some drugs, such as the currently used non-nucleoside reverse transcriptase inhibitors, have a low genetic barrier to resistance since only a single nucleotide mutation is required to completely loose drug activity. By contrast, other drugs (including most protease inhibitors) require an ordered accumulation of multiple mutations to confer resistance, and thus have a higher genetic barrier to resistance. At treatment failure, the virus is more likely to have developed resistance against the drug with the lower genetic barrier [4-6]. However, the actual genetic barrier is not merely the number of mutations needed to confer resistance, since the likelihood of a mutation is not uniform due to evolutionary restrictions. A mutation must also be considered in the context of *in vivo *fitness, reflecting the combination of phenotypic resistance and intrinsic replication capacity. Epistatic fitness interactions between mutations may alter the prevalence of a mutation depending on the presence of another mutation.

Genotypic resistance testing aims at uncovering mutational patterns in the virus and interpreting their impact on drug resistance. This interpretation is difficult because of the complexity of resistance patterns, the existence of cross-resistance and resensitization, and the high natural variation of HIV-1. Ideally, a genotypic resistance test not only helps in selecting a treatment regimen that will immediately inhibit viral replication, but also in selecting a treatment with a high genetic barrier to resistance, and thus a durable response. Therefore, not only the contribution to resistance of detected mutations, but also their impact on lowering the genetic barrier towards resistance should be considered. Because there is no readily available measure for the genetic barrier (unlike for the resistance phenotype, which may be measured using an *in vitro *assay), the impact of many mutations and mutational patterns on genetic barrier is not well understood. With a few exceptions, such as the so-called revertants at reverse transcriptase position 215 [7], the clinical relevance of a supposedly decreased genetic barrier has not been shown. A lower genetic barrier not only poses a higher long-term risk for failure in case of nonoptimal adherence, but may also impact treatment options available at failure, under the assumption that development of resistance at treatment failure is more likely for drugs with a lower genetic barrier, and because of the extensive cross-resistance within drug classes.

The extensive natural variation within the HIV-1 main group (reflected partly in subtype diversification) is not believed to impact drug susceptibility substantially [8,9]. Still, this variation may affect the genetic barrier to resistance for some drugs, even in treatment-naive patients, and this could in principle be predicted from the genotype [10]. Several studies have indeed suggested that the presence of polymorphisms, known as minor mutations, impact virological outcome [11-14]. However, these studies usually lacked statistical power to assign the effect on virological outcome to the presence of particular polymorphisms because of the small prevalence of many polymorphisms, and the confounding effect of adherence.

In previous work, we presented a method to estimate a fitness landscape experienced by the virus during treatment, and applied this in the context of the protease inhibitor nelfinavir (NFV) [15]. Simulated evolution from a baseline sequence, over such a fitness landscape, together with a criterion for resistance, allows the estimation of the individualized genetic barrier to resistance. In the present study, we investigate the association of the individualized genetic barrier with development of resistance at failure, as predicted by an expert rule-based genotypic interpretation system, in patients fully susceptible to NFV at baseline. We also explore genotypic factors that impact this estimated genetic barrier for viruses predicted to be fully susceptible to NFV.

## Results

### Predicting development of NFV resistance at treatment failure

The final longitudinal data set included 201 protease sequence pairs with a subtype distribution largely dominated by subtype B (78%). A Neighbor-Joining phylogenetic tree constructed from the baseline sequences revealed no intra-subtype clustering according to data source (data not shown). At treatment failure, the Rega algorithm predicted full NFV resistance (*R*), i.e. with GSS_NFV _= 0, in 73 cases (36%) and intermediate NFV resistance (*I*), i.e. with GSS_NFV _= 0.5, in 6 cases (3%).

In these pairs, genotypic susceptibility to NFV treatment as estimated *in vivo *fitness value and estimates of the simulated genetic barrier to resistance were computed from the baseline sequence (Table 1). Despite the fact that each patient was predicted at baseline to be fully susceptible to NFV by a genotypic interpretation system (Rega V8.0.1), we observed variation in estimated fitness under NFV treatment at baseline as well as substantial variation in estimated genetic barrier to NFV resistance (Table 1). The genotypic susceptibility of the virus to the remaining drugs in the combination, predicted by Rega, was high. For most patients (67%), the activity score for the combination excluding NFV (*GSS_Other_*) summed up to ≥ 2, which suggests that the majority of the NFV-based regimens was potent enough at the time of therapy initiation. The median time to treatment failure was 12 months.

**Table 1:** Descriptive characteristics of model variables

Factor	Characteristics
log	0.36 (0.2 - 0.6)
, mutations	2.7 (2.16 - 3.25)
, generations	114 (84 - 138)
Δ*T*, months	12 (6 - 23)
GSS_*Other*_	2 (1 - 3)
*R *= true, n (%)	76 (35%)
*Sub *= B, n (%)	157 (78%)

Description of model variables in a longitudinal data set for 201 patients, which were fully susceptible to NFV at baseline. Data are median (range) for log estimated fitness (), genetic barrier estimates ( and ), duration between baseline and follow-up sample (Δ*T*), the backbone activity (*GSS_Other_*) and the subtype distribution (*Sub*).

The results of the univariable analysis are shown in Table 2. A lower estimated genetic barrier, both in terms of mutations (OR = 0.65 (0.45 - 0.94) per additional mutation, p = .02) or in terms of generations (OR = 0.98 (0.97 - 0.99) per 10 more generations, p = .01) and lower activity of the other drugs in the combination (OR = 0.53 (0.39 - 0.71), p < 0.001) were associated with a higher risk of developing NFV resistance at treatment failure. Estimated fitness under NFV selective pressure, duration on therapy or subtype B virus were not associated with NFV resistance development.

**Table 2:** Univariable analysis of development of NFV resistance at failure

Variable	Odds ratio	95% CI	p Value
log , per unit higher	1.40	0.64 - 3.04	.39
, per additional mutation	0.65	0.45 - 0.94	.02
, per 10 generations more	0.98	0.97 - 0.99	.01
*GSS_Other_*, per unit higher	0.53	0.39 - 0.71	< .001
Δ*T*, per month more	1.00	0.99 - 1.01	.74
*Sub*, as B	1.63	0.83 - 3.22	.16

Univariable association of factors at baseline with risk of nelfinavir (NFV) resistance development at treatment failure: fitness under NFV treatment (log ), expected number of mutations to NFV resistance (), expected number of generations to NFV resistance (), time between baseline and follow-up sequence (Δ*T*), the activity of the other drugs in the combination (*GSS_Other_*) and the subtype distribution (*Sub*).

These significant associations remained in the multivariable analysis (Table 3). A lower genetic barrier in terms of mutations (OR = 0.54 (0.32 - 0.91) per additional mutation, p = .02) or in terms of generations (OR = 0.98 (0.97 - 0.99) per 10 more generations, p = .0075) associated significantly with an increased risk for developing NFV resistance at treatment failure. Also a lower backbone activity (OR = 0.49 (0.35 - 0.67), respectively p < .001 and p = .001) was independently indicative for acquiring NFV resistance.

**Table 3:** Multivariable analysis of development of NFV resistance at failure

Variable	Coefficient (*β*)	SE	P Value	Odds Ratio	95% CI
Intercept	1.68	1.27			
log , per unit higher	-0.51	0.56	.36	0.60	0.20 - 1.79
, per additional mutation	-0.61	0.26	.02	0.54	0.32 - 0.91
*GSS_Other_*, per unit higher	-0.72	0.17	< .001	0.49	0.35 - 0.67
Δ*T*, per month more	< 0.001	< 0.001	.14	1.00	0.99 - 1.01
*Sub*, as B	0.55	0.38	.15	1.73	0.82 - 3.64

**Variable**	**Coefficient (*β*)**	**SE**	**P Value**	**Odds Ratio**	**95% CI**

Intercept	3.31	1.44			
log , per unit higher	-0.67	0.56	.24	0.51	0.17 - 1.56
, per 10 generations more	-0.02	0.005	.008	0.98	0.97 - 0.99
*GSS_Other_*, per unit higher	-0.74	0.17	< .001	0.47	0.34 - 0.66
Δ*T*, per month more	< 0.001	< 0.001	.14	1.00	0.99 - 1.01
*Sub*, as B	0.41	0.38	.29	1.51	0.70 - 3.23

A multivariable logistic regression model is shown for development of nelfinavir (NFV) resistance at treatment failure starting from the baseline genotype based on the expected number of mutations to NFV resistance () in the upper table and based on the expected number of generations to NFV resistance () in the lower table. Analyses are corrected for duration between baseline and follow-up sequence (Δ*T*), fitness under NFV treatment (log ), the activity score of the combination excluding NFV (*GSS_Other_*) and the subtype distribution (*Sub*).

The two measures for genetic barrier were highly correlated (*R*^2 ^= 0.97 (0.96 - 0.98), p < 10^-16^), since each additional mutation in general requires extra evolutionary time to evolve. This also explains why the results were similar when using  versus .

### Genotypic correlates of estimated genetic barrier

To investigate contributions of protease mutations and polymorphisms on the predicted genetic barrier, evolution was simulated for a virtual cohort of 2764 patients on NFV treatment, and for each simulation, the number of generations *G_R _*to develop NFV resistance was recorded. Because the estimated fitness landscape only models intra-subtype variation for each subtype, this analysis was done only using HIV-1 subtype B, the most prevalent subtype in our data set. Phylogenetic reconstruction indicated interspersion of multiple lineages of sequences sampled in Portugal. Therefore, separation of sequences in the tree conditioned on the center of data collection could not be established (data not shown).

**Figure 1:**
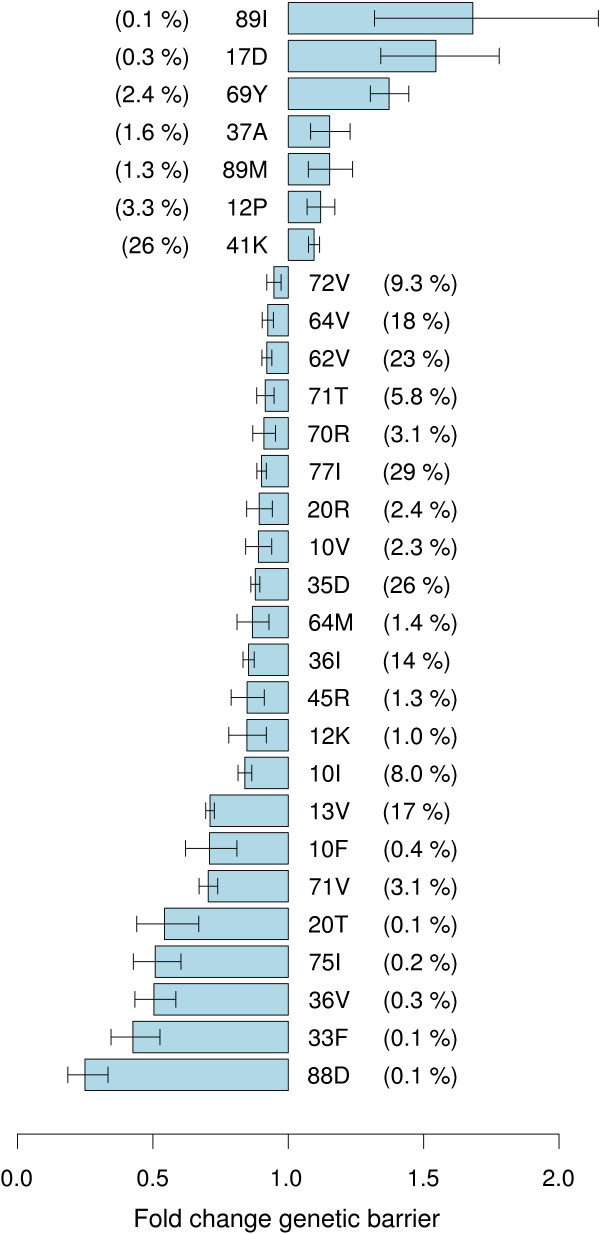
**Genotypic correlates of genetic barrier**. Impact of protease mutations and polymorphisms on the estimated genetic barrier to nelfinavir (NFV) resistance. For each mutation, the prevalence is indicated in the data set of protease inhibitor naive patients, which are all predicted as fully susceptible to NFV.

A step-wise model search was performed to identify a best linear model for log *G_R_*, which thus included the independent, multiplicative contributions of single mutations. In the final model, 22 mutations (10F/I/V, 12K, 13V, 20R/T, 33F, 35 D, 36I/V, 45R, 62V, 64M/V, 70R, 71T/V, 72V, 75I, 77I and 88D) independently decreased the genetic barrier (p < .05), while 7 mutations (12P, 17 D, 37A, 41K, 69Y and 89I/M) increased the genetic barrier (Figure 1). Although Figure 1 indicates contributions of pro-tease mutations to the genetic barrier with a fixed extent, these values resulted from averaging over the entire population (of 2764 sequences) and, since only independent and individual mutational contributions were considered, as well over mutations epistatically interacting with the respective mutation listed (see additional file 1 for the full model). As such, these findings do not contradict with the observation that the genetic context contributes to fitness in the landscape, and consequently to the genetic barrier to resistance. For example, mutation 71V was present in 85 isolates (3.1%), of which 45 (53%) selected 30N as first mutation and 9 (11%) 90 M (which are considered major resistance mutations by Rega). Baseline sequences lacking this mutation only selected in 487 (18%) and in 106 cases (4%) 30N and 90 M respectively. On the other hand, 9 isolates harboured mutation 17 D and 30N was only selected in 1 (11%) and 90 M (0%) zero cases, compared to 531 (19%) and 115 (4%) for isolates lacking 17 D.

For several of the mutations that contributed to a decreased genetic barrier (10V, 13V, 20R, 33F, 35 D, 36I/V, 45R, 62V, 64M/V, 70R, 71T/V, 77I, 88D) and one mutation that increased genetic barrier (89I), predicted selection by the fitness landscape model was shown previously to correlate with observed evolution in longitudinal data from patients on NFV treatment [15]. Thus, for these mutations, the fitness function modeled interactions with polymorphisms or other resistance mutations that affects their selection. Rega considers ten mutations (10I/V, 20R/T, 33F, 62V, 64V, 71T/V, and 88D) to contribute to resistance as minor mutations. Eleven mutations that were predicted to decrease genetic barrier (12K, 13V, 35 D, 36I/V, 45R, 64 M, 70R, 72V, 75I, and 77I), and five mutations that were predicted to increase the genetic barrier (12P, 17 D, 37A, 41K, 69Y, and 89M) are not included in the rules for NFV resistance in Rega. Some of these mutations have been described previously in relation to resistance to NFV or other protease inhibitors: mutations 36I and 77I are polymorphisms that are involved in NFV resistance [16]; mutation 45R has recently been associated with NFV treatment [17]; mutations 13V, 36I/V, 45R, 72V, 75I and 77I are associated with protease inhibitor treatment [18] and mutation 13V has been associated with reduced response to tipranavir [19]. Mutation 89I has been linked to treatment failure in several non-B subtypes, where the wild-type is 89 M [20]. 89I/M are rare mutations in subtype B, and the model indicates that in subtype B, they increase the genetic barrier to resistance because they are reverted to the wild type (89L), although the same model correctly predicts selection of 89I during NFV treatment in other subtypes [15].

## Discussion

In this study, we evaluated retrospectively the association of genotypic information contained in the baseline genotype with the risk of developing NFV resistance at treatment failure, when treated with a NFV containing regimen, in longitudinal sequence pairs. The baseline sequences were interpreted using an estimated fitness function for HIV-1 under NFV selective pressure, which was used to compute the estimated fitness (log ) and two measures of genetic barrier: the expected number of mutations  or generations  to evolve a mutational pattern that is considered by Rega as causing resistance to NFV. As expert resistance interpretation system, Rega was chosen because it has been clinically evaluated for prediction of treatment outcome [21]. This will allow us to investigate if the fitness landscape could be used to predict treatment options available at treatment failure as predicted by Rega.

Both in univariable and multivariable analyses, a lower genetic barrier was found to increase the risk for developing NFV resistance at treatment failure. Such estimated genetic barrier may provide unique and useful information to a clinician contemplated a change of treatment, allowing to take into account available therapy options in case of subsequent treatment failure. This is, to our knowledge, the first proof of direct clinical impact of (individualized) genetic barrier on risk of development of resistance at treatment failure.

With the goal of life-long treatment, options at treatment failure are taken into consideration at start of treatment, and therefore current HIV-1 treatment guidelines take into account proper drug sequencing and the sparing of inhibitor classes [22]. An individualized prediction of (cross-)resistance development at treatment failure may therefore contribute to a more informed treatment choice. Noteworthy, a lower activity of the regimen accompanying NFV, predicted by Rega, was associated with an increased risk of NFV resistance at therapy failure. This association can be expected since a suboptimal, less potent regimen may favor evolution and development of NFV resistance more easily. The accuracy of the predictions may be further improved by using the genetic barrier to resistance for the other drugs in the combination, instead of a susceptibility score.

The association of a lower genetic barrier with an increased risk for resistance development at failure implies indirectly that the estimated genetic barrier could also be predictive for long-term treatment response. Indeed, these results show that a lower genetic barrier facilitates resistance development, and may therefore be expected to increase as well the risk for treatment failure because of resistance development under non-optimal adherence. Although the Rega system for genotypic resistance interpretation also scores the presence of several minor resistance mutations as intermediate resistance (motivated by the principle that they may reduce the genetic barrier to resistance), in this analysis only patients were included for which Rega predicted full susceptibility to NFV at baseline. Assuming that viral fitness during treatment depends on the susceptibility of the virus to the drug, log  can be considered an *in vivo *resistance phenotype. The restriction of the study to patients predicted susceptible may explain why viral fitness, visualized by the virus position in the landscape, did not relate to the emergence of NFV resistance. Overall, these results provide additional indication that the estimated fitness landscape may outperform an expert system for prediction of treatment outcome, in particular for patients who are considered fully susceptible by the expert system [15].

Although clinical response in terms of viral load measurements was not available for these patients, the availability of a follow-up genotype is indicative of treatment failure. By requesting a genotypic test, the clinician presumed failure of the current regimen, and successful genotyping implied high enough viremia. We previously evaluated the performance of this landscape to predict virological outcome in a clinical cohort of patients, starting with a combination of zidovudine (AZT), lamivudine (3TC) and NFV. Differently from the current study, patients were not required to be fully susceptible to NFV. A higher genetic barrier was significantly associated with higher viral load reduction on short term and with lower odds of virological failure on long term [23].

For this analysis, sequence data were combined that originated from different geographic locations. Genotypic variation was accounted for by adding HIV-1 subtype to the model. Additionally, phylogenetic analysis did not unveil geographical withinsubtype sequence differences. By pooling data from multiple sources, (unknown) variables, besides epidemiology, could differ between patient groups and influence resistance development. The objective of the fitness estimation procedure was not to predict resistance development as such, but to quantify the influence of mutational patterns on viral fitness under drug selective pressure and eventually to predict virus evolution under this pressure. Resistant virus was defined by an independent interpretation algorithm. As measures of "time", we considered the number of mutations or simulated generations. The actual time to therapy failure is besides the evolutionary distance under drug selective pressure (quantified by the genetic barrier), a function of the rate at which HIV-1 will bridge this distance (quantified by the strength of drug selective pressure). Next to drug activity, the potency of treatment is the outcome of different parameters. Though information on patient-specific parameters (such as therapy adherence) or on management of HIV-1 infection is missing, these parameters most likely do not influence the actual evolutionary distance to resistance, but do affect drug potency and subsequently the time to therapy failure. Hence, the time between therapy initiation and failure was included as a variable to correct for (hidden) variables that influence the amount of virus evolution tolerated.

To obtain an insight into the contributions of mutations and polymorphisms towards estimated genetic barrier to NFV resistance in isolates susceptible to NFV, we simulated resistance evolution during NFV treatment in subtype B sequences from a large virtual clinical patient cohort. A total of 29 mutations and polymorphisms were identified that independently contribute to the genetic barrier to NFV resistance (Figure 1).

Because of the combined use of the fitness landscape with an expert system, a mutation may influence the estimated genetic barrier either because it contributes to resistance (as predicted by Rega), or because it influences, in the fitness function, the selection of mutations that contribute to resistance, or both. A number of mutations (12K/P, 13V, 17 D, 35 D, 36I/V, 37A, 41K, 45R, 64 M, 69Y, 70R, 72V, 75I, 77I and 89M) are not included in the rules for NFV resistance. Therefore, each of these mutations contributes to a lower (respectively higher) estimated genetic barrier through their inclusion in the fitness landscape model, where they cause a faster (respectively slower) selection of resistance mutations that are considered by Rega. The mechanism for the contribution to a lower genetic barrier of mutations (10I/V, 20R/T, 33F, 62V, 64V, 71T/V, and 88D) which are considered by Rega to contribute to resistance (as minor resistance mutation), may be because of their inclusion in the rule for predicting resistance in Rega, or because of an influence on selection of (major) resistance mutations in the fitness landscape model, or both.

The contribution of a mutation to viral fitness is highly dependent on the genetic background, and a mutation with an impact on the genetic barrier was identified by the model conditioned on the presence of polymorphic variation. Despite the recruitment of only subtype B sequences, and phylogenetic analysis that indicated distributed sequences among the tree, intra-subtype variation, as a consequence of founder effects, is inevitable and has also been reported [24]. A total of 10 mutations listed in Figure 1 differed significantly (p < 0.05) in prevalence between the two patients groups (see additional file 2). However, these mutations still contributed significantly to the genetic barrier when the analysis was restricted to data source, highlighting the role of sequence variability. Application of the same methodology to another subtype B dataset may conceivably not identify exactly the same set of mutations, given that genotypic (geographical) variation exists within a subtype. These findings argue the usefulness of the genetic barrier to predict resistance development, and the influence of the genetic background on this parameter. Knowledge extracted from this analysis could be used to enhance prediction of therapy outcome.

As evolutionary simulator of the HIV-1 intrahost population, an ideal Wright-Fisher model of molecular evolution was assumed, which is a well accepted model for evolution in a finite population. A number of assumptions were implemented to reduce the (computational) complexity of the model (see additional file 3). The model did not include recombination. These simplifications may be avoided with availability of a more accurate, but also more computationally demanding simulator. Although recombination can speed up resistance accumulation, the fitness landscape attempts to capture the selective advantage of mutational patterns under drug selective pressure, what is not expected to be influenced by recombination.

## Conclusions

In conclusion, we have demonstrated for the first time the existence of intra-patient variation in genetic barrier to resistance (in this study, to nelfinavir) in patients considered fully susceptible by an expert system. The estimated genetic barrier not only reflects the amount of genetic change needed for resistance, but also takes into account the influence of virus genetic background, evolutionary constraints as well as the relative impact of a mutation on the *in vivo *fitness. We found that a lower individualized genetic barrier was associated with a higher risk for development of resistance at treatment failure. The genetic barrier to resistance, estimated at baseline, may uncover more information predictive for developing resistance than currently used genotypic algorithms.

## Methods

### Clinical data

Clinical data was pooled from the Stanford HIV Drug Resistance Database [25] and from a clinical database maintained at the Molecular Biology Laboratory of Centro Hospitalar de Lisboa Occidental, on behalf of the Portuguese HIV Resistance Study Group.

To investigate a correlation between estimated genetic barrier to NFV resistance and development of NFV resistance at failure, patients were selected that failed on a NFV as first containing treatment, and for whom a protease sequence was available both at start of treatment and at treatment failure. Patients did not have previous PI history. The activity of NFV at baseline and failure was predicted using the Rega v8.0.1 algorithm for genotypic resistance interpretation [21]. Only patients with full genotypic susceptibility to NFV at baseline (Genotypic Susceptibility Score (GSS_NFV _= 1) were included, and at most one sequence pair per patient. None of these sequences were included in the data used to estimate the fitness function under NFV selective pressure. Genotypic susceptibility (*GSS_Other_*) for the therapy combination (excluding NFV) was computed by summing up the individual GSS of the other drugs in the combination. HIV-1 subtype distribution of the population was determined from the protease and partial reverse transcriptase sequences using the REGA HIV-1 Subtype tool v2.0 [26]. Isolates were classified as either subtype B or nonB (*Sub*), as B was the majority subtype in the longitudinal data set.

To investigate genotypic correlates in protease with the estimated genetic barrier to NFV resistance, evolution under NFV treatment was simulated for a large cohort of susceptible protease sequences (with GSS_NFV _= 1). At most one sequence per patient was used. The technique of fitness landscape was developed to particularly take into account the large natural diversity of HIV-1, in order to estimate a fitness landscape that could be used across subtypes. However, since the method relies on within patients evolution towards higher fitness under drug selective pressure, evolution from one subtype to another, even if more fit under treatment, will never be observed. Therefore, resistance evolution can never capture fitness differences between subtypes, and thus, *in vivo *fitness and derived genetic barrier are directly comparable only within each subtype individually, but not across subtypes [15]. To avoid a subtype bias, only subtype B sequences were included.

### NFV fitness function

A fitness landscape of HIV-1 under NFV selective pressure was previously estimated from cross-sectional data, as described in detail in [15] (an overview of the methodology and a list of mutations included in the fitness function can be found in additional file 3). Briefly, we estimated a fitness function compatible with differences in prevalence of mutational patterns observed in sequences from untreated and treatment experienced patients that are the result of convergent evolution under selective pressure modeled by the fitness function. More specifically, we contrasted 7774 sequences obtained from protease inhibitor naive patients with 1026 sequences from patients treated with NFV as single protease inhibitor. These sequences were of diverse subtypes (B: 66%, G: 15%, C: 7% and other: 13%).

Estimated fitness is based on the assumption that when a mutation, or pattern of mutations, is independently fixed in a population under selective pressure of the same treatment in multiple patients, this convergent evolution may indicate that the mutation or pattern increases the fitness of the virus in that environment. Since an interaction between two mutations is expected to lead to a different observed prevalence of one mutation depending on the presence of the other, conditional dependencies in mutations prevalence (identified by Bayesian Network Learning) may indicate epistatic fitness interactions between these mutations [27]. These interactions are incorporated in a multiplicative fitness function, which describes fitness as a product of independent contributions of presence of 114 amino mutations at 48 protease positions, augmented with independent contributions for combinations of interacting mutations. So the fitness contribution of a mutation is dependent on the presence of mutations with which it interacts.

To estimate fitness function parameters, the fitness function was combined with a simulator of HIV-1 intra-host evolution, making the connection between naive protease sequences, treatment selective pressure, and sequences from patients failing treatment. The fitness landscape was scaled so that fitness of the HIV-1 subtype B reference strain HXB2 was 1, so that for any given sequence put in the landscape, a fitness number is computed that represents the relative fitness compared to HXB2.

### Correlation of estimated genetic barrier to NFV resistance with resistance development at treatment failure

The NFV fitness landscape was used to estimate, for each baseline sequence of 201 longitudinal pairs, viral fitness under NFV treatment and the simulated genetic barrier to NFV resistance. The position of the viral sequence in the landscape can be considered as quantification of genotypic susceptibility. For a baseline sequence, this predicted viral fitness under NFV treatment () (fitness number as explained above and expressed in log scale) was computed as the average fitness of 100 baseline sequences, in which nucleotide mixtures were removed from each sequence by random sampling one of the pure nucleotides from the mixture.

The genetic barrier to NFV resistance for a sequence was calculated by simulating HIV-1 evolution using the estimated fitness landscape and the simulator of HIV-1 intra-host evolution [15]. For each sequence, the genetic barrier was quantified as the average number of mutations () or number of simulated generations () until full resistance was predicted by Rega (GSS_NFV _= 0) in 100 evolution simulation runs. At the start of each simulation, nucleotide mixtures were removed as described before.

The associations of log ,  and  with odds for development of NFV resistance *R *at failure, were investigated using univariable and multivariable logistic regression models. Variables included in the multivariable models were log ,  or  (per 10 generations), duration between baseline and follow-up sample, *GSS_Other _*and subtype distribution (*Sub*).

### Identifying genotypic correlates of estimated genetic barrier

To investigate genotypic correlates of the estimated genetic barrier to NFV resistance, evolution under NFV treatment was simulated for a virtual clinical cohort of 2764 patients fully susceptible to NFV at baseline (GSS_NFV _= 1). For each sequence, one simulation run was performed using the fitness landscape and the number of simulated generations *G_R _*was recorded until full resistance was predicted by Rega.

Subsequently, a step-wise linear model selection was performed for log *G_R_*, in order to investigate the independent multiplicative contributions of the presence of individual mutations to the estimated genetic barrier. The model selection started from an empty model, and considered all 114 mutations that were included in the fitness function model. At each step, the addition to or removal from the model of each mutation was considered, and the change that resulted in a model with lowest Akaike Information Criterion (AIC) score was selected, until no more improvement was observed. All statistical analyses were performed using R 2.2.1 [28].

## Authors' contributions

KT, KD and GB performed the analyses. KD designed and implemented the analysis software. RJC, EvW, SYR and RWS contributed clinical and virological data. PL, KvL and A-MV contributed to discussions and A-MV supervised the work. All coauthors contributed to the design of the study and interpretation of the results. All authors have read and approved the final manuscript.

## Supplementary Material

Additional file 1 **Genotypic correlates of estimated genetic barrier**. A step-wise linear model selection procedure was performed to investigate the independent, multiplicative contributions of presence of individual, baseline mutations to the genetic barrier. The analysis yielded in total 43 mutations, of which 29 were significantly associated. Columns denote baseline mutation, estimated coefficient, standard error, t-statistic and corresponding (two-sided) p-value of the fitted model. Click here for file

Additional file 2 **Distribution of mutation prevalence between collection centers**. For each of the 114 protease mutations included in the fitness function, the prevalence and corresponding percentage are shown with respect to the database from which data was pooled, as well as the p-value, odds ratio (OR) and the adjusted p-value using Bonferroni correction for multiple testing. Data were retrieved from either a database maintained at the Molecular Biology Laboratory of Centro Hospitalar de Lisboa Occidental in Portugal (PT) or from the Stanford HIV Drug Resistance Database (HIVDB). A total of 19 mutations differed significantly in prevalence between the two patient groups. Mutations that significantly contributed to the genetic barrier to NFV resistance (listed in Figure 1) are indicated in bold. Click here for file

Additional file 3 **Estimating a HIV-1 fitness landscape under selective pressure**. A brief overview of the method to estimate an in vivo fitness landscape experienced by HIV-1 under drug selective pressure, from observed evolution in clinical sequences. Click here for file
