# Supplementary material for: Estimating the individualized HIV-1 genetic barrier to resistance using a nelfinavir fitness landscape
Source: BMC Bioinformatics. 2010 Aug 3;11:409. doi: 10.1186/1471-2105-11-409 (PMC2921410; doi:10.1186/1471-2105-11-409)
Supplement: Additional file 1 — Genotypic correlates of estimated genetic barrier. A step-wise linear model selection procedure was performed to investigate the independent, multiplicative contributions of presence of individual, baseline mutations to the genetic barrier. The analysis yielded in total 43 mutations, of which 29 were significantly associated. Columns denote baseline mutation, estimated coefficient, standard error, t-statistic and corresponding (two-sided) p-value of the fitted model. [file 1471-2105-11-409-S1.PDF]

Var	Estimate	Std. Error	t value	Pr(>|t|)
13V	-0.34	0.02	-15.25	1.99E-050
71V	-0.35	0.05	-7.22	6.52E-013
35D	-0.13	0.02	-6.88	7.27E-012
36I	-0.16	0.02	-6.56	6.21E-011
69Y	0.32	0.05	6.13	9.92E-010
10I	-0.18	0.03	-5.78	8.39E-009
77I	-0.1	0.02	-5.36	9.03E-008
41K	0.09	0.02	4.88	1.12E-006
88D	-1.39	0.29	-4.72	2.48E-006
36V	-0.69	0.15	-4.6	4.47E-006
62V	-0.08	0.02	-4.13	3.72E-005
33F	-0.85	0.21	-4.05	5.16E-005
75I	-0.68	0.17	-3.94	8.47E-005
64V	-0.08	0.02	-3.57	3.69E-004
17D	0.44	0.14	3.08	2.07E-003
20T	-0.61	0.21	-2.92	3.54E-003
10F	-0.34	0.13	-2.58	9.85E-003
71T	-0.09	0.04	-2.54	1.11E-002
12P	0.11	0.05	2.47	1.37E-002
45R	-0.17	0.07	-2.29	2.24E-002
37A	0.14	0.06	2.25	2.48E-002
10V	-0.12	0.05	-2.17	3.01E-002
20R	-0.11	0.05	-2.14	3.21E-002
89I	0.52	0.24	2.13	3.30E-002
64M	-0.14	0.07	-2.09	3.64E-002
70R	-0.09	0.05	-2.04	4.10E-002
12K	-0.17	0.08	-2.03	4.23E-002
89M	0.14	0.07	2	4.55E-002
72V	-0.06	0.03	-1.97	4.84E-002
57K	-0.05	0.02	-1.86	6.32E-002
37C	0.11	0.06	1.86	6.35E-002
46I	-0.28	0.15	-1.84	6.61E-002
19V	-0.12	0.06	-1.91	5.62E-002
12A	0.08	0.04	1.82	6.85E-002
33V	0.08	0.05	1.77	7.76E-002
69Q	0.08	0.05	1.8	7.25E-002
70E	-0.24	0.14	-1.7	8.98E-002
93L	0.03	0.02	1.63	1.04E-001
69K	0.1	0.06	1.62	1.05E-001
65D	0.08	0.05	1.62	1.06E-001
63P	-0.03	0.02	-1.61	1.07E-001
19I	0.05	0.03	1.63	1.07E-001
12I	-0.11	0.07	-1.51	1.32E-001
