# Supplementary material for: Estimating the individualized HIV-1 genetic barrier to resistance using a nelfinavir fitness landscape
Source: BMC Bioinformatics. 2010 Aug 3;11:409. doi: 10.1186/1471-2105-11-409 (PMC2921410; doi:10.1186/1471-2105-11-409)
Supplement: Additional file 2 — Distribution of mutation prevalence between collection centers. For each of the 114 protease mutations included in the fitness function, the prevalence and corresponding percentage are shown with respect to the database from which data was pooled, as well as the p-value, odds ratio (OR) and the adjusted p-value using Bonferroni correction for multiple testing. Data were retrieved from either a database maintained at the Molecular Biology Laboratory of Centro Hospitalar de Lisboa Occidental in Portugal (PT) or from the Stanford HIV Drug Resistance Database (HIVDB). A total of 19 mutations differed significantly in prevalence between the two patient groups. Mutations that significantly contributed to the genetic barrier to NFV resistance (listed in Figure 1) are indicated in bold. [file 1471-2105-11-409-S2.PDF]

Var	PT	%	HIVDB	%	P-value	OR	Adjusted P
57K	115	26.1	226	9.71	2.1E-18	3.29	2.4E-16
37E	42	9.5	40	1.72	4.4E-14	6.03	2.6E-12
36L	35	8.0	27	1.16	1.4E-13	7.35	5.5E-12
37D	90	20.5	226	9.71	1.4E-09	2.39	4.0E-08
14R	88	20.0	261	11.22	1.5E-06	1.98	3.4E-05
**41K**	151	34.3	558	23.98	9.8E-06	1.66	1.9E-04
20I	8	1.8	3	0.13	4.1E-05	14.32	0.001
93L	159	36.1	615	26.43	4.8E-05	1.57	0.001
**36I**	86	19.5	298	12.81	3.0E-04	1.65	0.004
63L	94	21.4	685	29.44	5.1E-04	0.65	0.006
**12P**	27	6.1	64	2.75	6.9E-04	2.31	0.007
**77I**	156	35.5	638	27.42	8.4E-04	1.45	0.008
**20R**	21	4.8	45	1.93	9.9E-04	2.54	0.009
**13V**	97	22.0	362	15.56	1.3E-03	1.53	0.010
**71V**	25	5.7	60	2.58	1.3E-03	2.28	0.010
**10I**	19	4.3	202	8.68	1.5E-03	0.47	0.010
**62V**	126	28.6	504	21.66	1.9E-03	1.45	0.013
**35D**	140	31.8	575	24.71	2.4E-03	1.42	0.015
58E	5	1.1	4	0.17	0.01	6.67	0.043
**37A**	14	3.2	31	1.3	0.01	2.4	0.07
**64V**	62	14.1	441	19.0	0.02	0.7	0.08
67S	7	1.6	13	0.6	0.03	2.9	0.15
**89M**	11	2.5	26	1.1	0.04	2.3	0.19
33V	8	1.8	88	3.8	0.05	0.5	0.22
15V	87	19.8	369	15.9	0.05	1.3	0.23
72T	23	5.2	77	3.3	0.05	1.6	0.23
**45R**	10	2.3	25	1.1	0.06	2.1	0.25
**12K**	8	1.8	19	0.8	0.06	2.2	0.25
93M	2	0.5	1	0.0	0.07	10.6	0.27
63A	13	3.0	110	4.7	0.10	0.6	0.39
37H	3	0.7	41	1.8	0.14	0.4	0.53
85V	1	0.2	0	0.0	0.16	Inf	0.57
**10V**	14	3.2	49	2.1	0.16	1.5	0.57
**70R**	9	2.0	77	3.3	0.18	0.6	0.60
**71T**	32	7.3	129	5.5	0.18	1.3	0.60
46L	3	0.7	7	0.3	0.20	2.3	0.65
72E	2	0.5	29	1.2	0.21	0.4	0.67
39S	14	3.2	50	2.1	0.22	1.5	0.68
72M	8	1.8	26	1.1	0.24	1.6	0.69
18H	4	0.9	10	0.4	0.26	2.1	0.74
67E	1	0.2	1	0.0	0.29	5.3	0.80
41N	1	0.2	1	0.0	0.29	5.3	0.80
17E	10	2.3	36	1.5	0.31	1.5	0.81
37K	2	0.5	5	0.2	0.31	2.1	0.81
19V	5	1.1	44	1.9	0.33	0.6	0.82
82I	11	2.5	41	1.8	0.34	1.4	0.82
63V	1	0.2	18	0.8	0.34	0.3	0.82
33I	5	1.1	16	0.7	0.36	1.7	0.82

Var	PT	%	HIVDB	%	P-value	OR	Adjusted P
63T	22	5.0	94	4.0	0.36	1.3	0.82
61N	0	0.0	9	0.4	0.37	0.0	0.82
70E	0	0.0	9	0.4	0.37	0.0	0.82
**36V**	0	0.0	8	0.3	0.37	0.0	0.82
**89I**	1	0.2	2	0.1	0.41	2.6	0.88
65D	9	2.0	67	2.9	0.43	0.7	0.91
19T	7	1.6	28	1.2	0.49	1.3	0.97
63S	27	6.1	123	5.3	0.49	1.2	0.97
61E	12	2.7	52	2.2	0.49	1.2	0.97
63H	8	1.8	59	2.5	0.50	0.7	0.97
35N	1	0.2	3	0.1	0.50	1.8	0.97
19Q	9	2.0	37	1.6	0.54	1.3	1.00
19I	35	8.0	166	7.1	0.55	1.1	1.00
92K	2	0.5	20	0.9	0.56	0.5	1.00
16E	17	3.9	77	3.3	0.57	1.2	1.00
34D	1	0.2	4	0.2	0.58	1.3	1.00
74A	1	0.2	4	0.2	0.58	1.3	1.00
12A	19	4.3	88	3.8	0.59	1.1	1.00
64L	20	4.5	93	4.0	0.60	1.1	1.00
46I	2	0.5	6	0.3	0.62	1.8	1.00
**17D**	2	0.5	7	0.3	0.64	1.5	1.00
37S	88	20.0	443	19.0	0.64	1.1	1.00
37T	16	3.6	75	3.2	0.66	1.1	1.00
**10F**	2	0.5	8	0.3	0.67	1.3	1.00
12S	17	3.9	80	3.4	0.67	1.1	1.00
63Q	7	1.6	46	2.0	0.71	0.8	1.00
37C	7	1.6	46	2.0	0.71	0.8	1.00
69Q	16	3.6	78	3.4	0.77	1.1	1.00
12I	4	0.9	29	1.2	0.81	0.7	1.00
60E	29	6.6	146	6.3	0.83	1.1	1.00
43R	9	2.0	54	2.3	0.86	0.9	1.00
**69Y**	11	2.5	57	2.4	0.87	1.0	1.00
**64M**	6	1.4	33	1.4	1.00	1.0	1.00
71I	0	0.0	2	0.1	1.00	0.0	1.00
69K	7	1.6	36	1.5	1.00	1.0	1.00
61H	2	0.5	12	0.5	1.00	0.9	1.00
20M	2	0.5	10	0.4	1.00	1.1	1.00
20V	0	0.0	0	0.0	1.00	1.0	1.00
23I	0	0.0	1	0.0	1.00	0.0	1.00
61D	1	0.2	5	0.2	1.00	1.1	1.00
15L	1	0.2	7	0.3	1.00	0.8	1.00
66F	0	0.0	0	0.0	1.00	1.0	1.00
16A	3	0.7	18	0.8	1.00	0.9	1.00
63R	1	0.2	7	0.3	1.00	0.8	1.00
63C	4	0.9	23	1.0	1.00	0.9	1.00
54V	0	0.0	0	0.0	1.00	1.0	1.00
**88D**	0	0.0	2	0.1	1.00	0.0	1.00
73S	0	0.0	1	0.0	1.00	0.0	1.00

Var	PT	%	HIVDB	%	P-value	OR	Adjusted P
12N	4	0.9	25	1.1	1.00	0.8	1.00
82A	0	0.0	0	0.0	1.00	1.0	1.00
74K	0	0.0	0	0.0	1.00	1.0	1.00
74S	0	0.0	2	0.1	1.00	0.0	1.00
92R	1	0.2	5	0.2	1.00	1.1	1.00
89T	0	0.0	0	0.0	1.00	1.0	1.00
88S	0	0.0	0	0.0	1.00	1.0	1.00
90M	0	0.0	0	0.0	1.00	1.0	1.00
**33F**	0	0.0	4	0.2	1.00	0.0	1.00
35G	0	0.0	2	0.1	1.00	0.0	1.00
39Q	2	0.5	15	0.6	1.00	0.7	1.00
30N	0	0.0	0	0.0	1.00	1.0	1.00
72L	2	0.5	10	0.4	1.00	1.1	1.00
72V	41	9.3	218	9.4	1.00	1.0	1.00
89V	0	0.0	0	0.0	1.00	1.0	1.00
**75I**	1	0.2	5	0.2	1.00	1.1	1.00
45Q	0	0.0	2	0.1	1.00	0.0	1.00
**20T**	0	0.0	4	0.2	1.00	0.0	1.00
