# Supplementary material for: Estimating the individualized HIV-1 genetic barrier to resistance using a nelfinavir fitness landscape
Source: BMC Bioinformatics. 2010 Aug 3;11:409. doi: 10.1186/1471-2105-11-409 (PMC2921410; doi:10.1186/1471-2105-11-409)
Supplement: Additional file 3 — Estimating a HIV-1 fitness landscape under selective pressure. A brief overview of the method to estimate an in vivo fitness landscape experienced by HIV-1 under drug selective pressure, from observed evolution in clinical sequences. [file 1471-2105-11-409-S3.PDF]

ESTIMATING A FITNESS LANDSCAPE EXPERIENCED BY HIV-1 UNDER SELECTIVE PRESSURE

The estimates for the individualized genetic barrier to NFV resistance, computed for this study, were derived by simulation of HIV-1 evolution over a fitness landscape. A detailed and complete description of the estimation of this fitness model, as well as the evaluation of its performance to predict evolution are nicely illustrated in Deforche *et al.* (2008).

Briefly, a computational method was used to estimate the *in vivo* HIV-1 fitness function (F) under NFV selective pressure. The estimated fitness landscape reflects the selective pressures on HIV-1 to relate differences in prevalence of mutations and patterns of mutations in patient failing NFV treatment compared to naive patients with the selective advantage of these mutations or patterns. To learn a function $F(A_1, \ldots, A_n)$, where A_i presents presence or absence of a mutation, we find a function that fits with the evolution of the virus in a naive population of patients $\mathcal{P}^N$ to a treated population $\mathcal{P}^T$, and is closest to neutrality (minimizing $|F - 1|$).

Estimated fitness was based on the evolutionary principle that substitutions observed in the consensus sequence of a population under strong selective pressure are mostly fixed to increase the fitness of the population. As such, the increase in prevalence of a particular mutation in the population of sequences after failure, compared to the population of sequences that were naive, reflects the consecutive fixation of mutations in a population that acquires increased fitness under selective pressure. Not only increase in prevalence of individual mutations was considered, but also of patterns of mutations since epistatic fitness interactions alter the fitness impact of mutations depending on a context of other mutations. An interaction between two mutations is expected to lead to a different observed prevalence of one mutation depending on the presence of the other, observed associations in prevalence may indicate such fitness interactions. The fitness function F incorporates interactions indicated using Bayesian network (BN) learning, and its parameters are estimated using an iterative procedure where evolution for $\mathcal{P}^N$ over the current fitness function estimate is simulated, and compared to $\mathcal{P}^T$.

Fitness function structure

The protease amino acid sequences from the treated population $\mathcal{P}^T$ were used to learn interactions between mutations as described before (Deforche *et al.*, 2006). Briefly, a data set was created where a boolean variable indicated the presence of each included mutation. BN structure learning (Myllymäki *et al.*, 2002) on this boolean data was used to discover relationships between these mutations that may indicate epistatic fitness effects. By assuming conditional independencies, the Bayesian network refactors the Joint Probability Distribution (JPD) in a product of Conditional Probability Distributions (CPD), leading to a reduction in number of parameters to model the JPD. Formally, for n variables $A_1, \ldots, A_n$ (representing amino acid mutations), we would write:

$$P(A_1, \ldots, A_n) = \prod_i^n P(A_i|\text{parents}(A_i))$$

with $P(A|B)$ the conditional probability of A given B, and $\text{parents}(A_i)$ the parents in the BN structure of variable A_i. We denote the most probable network of the amino acid sequences of the treated population $\mathcal{P}^T$ with structure S^T and CPD parameters θ^T as $BN^T(\theta^T, S^T)$.

We model the relative fitness function $F(A_1, \ldots, A_n)$ in the same way as $BN^T(\theta^T, S^T)$ refactors the JPD:

$$F(A_1, \ldots, A_n) = \prod_i^n F(A_i|\text{parents}(A_i))$$

with $\text{parents}(A_i)$ the parents in S^T, and $F(A|B)$ the *Conditional Fitness Contribution (CFC)* of the presence of A, depending on the presence of B. The assumption here is that if two mutations are synergistic for example, they would occur more often together than not, and a dependency should be visible in the JPD too.

The CPDs are modeled by specifying the probability for a mutation A_i given any pattern of parent mutations k, in Conditional Probability Tables (CPTs): $\theta_{i,k} = P(A_i = 1 \,|\, \text{parents}(A_i) = k)$. Similarly, we used Conditional Fitness Tables (CFTs) to model the CFCs for each mutation A_i, which specify a different fitness contribution of the presence of a mutation A_i for every pattern of parent mutations: $\phi_{i,k} = F(A_i = 1 \,|\, \text{parents}(A_i) = k)$.

Example of Bayesian network and corresponding Fitness landscape structure

A hypothetical Bayesian Network structure shown in the Figure refactors the JPD describing presence of three mutations (30N, 88D, and 90M) as follows:

$$P(30\text{N}, 88\text{D}, 90\text{M}) = P(30\text{N})P(88\text{D}|30\text{N})P(90\text{M})$$

The corresponding relative fitness function $F(30\text{N}, 88\text{D}, 90\text{M})$ is then:

$$F(30\text{N}, 88\text{D}, 90\text{M}) = F(30\text{N})F(88\text{D}|30\text{N})F(90\text{M})$$

where F(30N) represents a fitness contribution of mutation 30N, and F(88D—30N) represents a fitness contribution of mutation 88D depending on presence of mutation 30N. Thus, mutations 30N and 90M contribute independently to fitness, while the fitness contribution of 88D is dependent on the presence of 30N. The values of these contributions are not simply based on the parameters of the Bayesian Network, but instead estimated taking also into account the prevalence of mutations in treatment naive patients and a model of evolution during treatment.

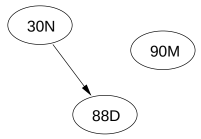

Fig. 1. Hypothetical Bayesian network for a data set with three mutations: 30N, 88D and 90M. The network structure indicates that 90M occurs independently from 30N or 88D, and a conditional prevalence of 88D on 30N.

Table 1. Protease mutations included in the fitness function

pos	wildtype	mutations	pos	wildtype	mutations	pos	wildtype	mutations	pos	wildtype	mutations
10	L	V,F,I	33	L	V,F,I	58	Q	E	72	I	E,L,V,M,T
12	T	K,P,N,I,S,A	34	E	D	60	D	E	73	G	S
13	I	V	35	E	G,N,D	61	Q	D,E,N,H	74	T	A,S,K
14	K	R	36	M	V,I,L	62	I	V	75	V	I
15	I	V,L	37	N	S,K,H,E,D,A,T,C	63	P	L,S,C,T,H,R,Q,V,A	77	V	I
16	G	A,E	39	P	S,Q	64	I	L,M,V	82	V	A,I
17	G	D,E	41	R	K,N	65	E	D	85	I	V
18	Q	H	43	K	R	66	I	F	88	N	S,D
19	L	V,T,I,Q	45	K	R,Q	67	C	E,S	89	L	T,V,M,I
20	K	V,R,M,T,I	46	M	I,L	69	H	K	90	L	M
23	L	I	54	I	V	70	K	E,R	92	Q	K,R
30	D	N	57	R	K	71	A	T,I,V	93	I	L,M

wildtype and mutations at protease positions included in the study. The most prevalent amino acid at each position was considered the wildtype, which corresponded mostly to the consensus subtype B sequence. Presence of the wildtype amino acid was not included as a separate variable, but was indicated by the absence of any of the included mutations. The fitness function was modelled based on presence of each of the mutations.

Model of evolution

A model of evolution was implemented that describes evolution in a finite population over a fitness landscape modeling a specific selective pressure. The evolutionary model is based on the Wright-Fisher model of evolution. The Wright-Fisher model describes evolution in a finite population by assuming discrete generations and that the probability for an individual to give offspring in the next generation is proportional to its fitness. After selection, a Poisson process is assumed for mutation at each locus. In addition, we assumed a constant population size N_e (the effective population size of the HIV-1 intra-host population).

The fitness function $F(N)$ that is used by the model, describes fitness of a nucleotide sequence N only as a function of the encoded amino acid sequence, even though the model implements evolution of nucleotide sequences. The implemented model does not detail evolution for each individual virus in the population separately, but considers only evolution of the population as a whole, and models fixations of single nucleotide mutations in the consensus nucleotide sequence N of this population. The model allows to obtain a sample $m_1, \ldots, m_n$ of n consecutive nucleotide fixations, from the distribution $P(M_1, \ldots, M_n|N_0)$ of n consecutive nucleotide substitutions that are expected given a population with initial consensus sequences N_0. Furthermore, the model assumes that fixation of the next mutation only depends on the current nucleotide consensus sequence, and not on previous states. Therefore,

$$P(M_1, \ldots, M_n|N_0) = P(M_1|N_0)P(M_2|N_1)\ldots P(M_n|N_{n-1})$$

with $N_i = M_i(N_{i-1})$, the nucleotide sequence obtained after substitution of mutation M_i in the sequence N_{i-1}.

At each step, the Wright-Fisher model was used to sample from $P(M|N)$, the distribution of the next expected mutation M given the current consensus sequence N assuming a fitness function F.

The computation uses (1) the fitness of the current consensus sequence, $F(N)$, and of the K sequences $F(m_k(N))$ which are in the one-nucleotide sequence neighbourhood of the current consensus sequence; (2) μ_k, the nucleotide mutation rate for each mutation m_k; and (3) N_e, the effective population size (see Figure 2). The nucleotide mutation rate is the rate at which new mutations arise during each replication cycle, which is independent of the selective pressure (Deforche *et al.*, 2007). From $F(N)$ and $F(m_k(N))$, the selective advantage s_k for each mutation m_k was computed:

$$s_k = \frac{F(m_k(N))}{F(N)} - 1.$$

In the real intra-host HIV-1 population, each of these mutations will be generated at rate μ_k simulateneously and all K alleles $m_k(N)$ compete with each other and with the current consensus sequence N for fixation. Unfortunately, the problem of determining the distribution of fixation probabilities for the K-allele problem is mathematically intractable (Ewens, 1979), and simulation prohibitively time consuming. Instead, the K-allele problem was approximated by considering K times a 2-allele problem. For each mutation m_k, a sample t_k was drawn from the distribution of population 50% fixation times $T_{50}(N_e, s_k, \mu_k)$ (in number of generations) of an allele with mutation m_k, starting from a population with 100% N alleles, where $m_k(N)$ was generated (and lost) at rate μ_k. The mutation k with the minimum sampled 50% fixation time t_k was used as an approximation for a sample drawn from the distribution of mutations that reached 50% fixation in the K-allele problem. No mathematical expression is known for $T_{50}(N_e, s, \mu_k)$, the time until the frequency of a mutant allele rises to 50% for the two-allele problem (Wang and Rannala, 2004). We found that this distribution could be reasonably approximated by a shifted log normal distribution

$$P(t; a, \mu, \sigma) = \frac{1}{(t-a)\sigma\sqrt{2\pi}} e^{-(\ln t - a - \mu)^2/2\sigma^2}$$

with parameters a, μ and σ obtained by fitting to 50% fixation times obtained from simulating the Wright-Fisher model with mutation and selection (see Figure 3). We could not use the 100% fixation time, since in presence of a non-zero mutation rate, back-mutation prevents fixation up to 100%. A threshold of 50% was chosen instead since the HIV-1 sequence datasets are obtained through population sequencing which can detect mutations if present at 50%.

Given our fitness function model, which allows variation in fitness based only on a subset of the full set of 20 amino acids at all positions, only mutations resulting in the evolution over this fitness landscape were considered: synonymous nucleotide mutations, or nucleotide mutations that resulted either in an amino acid change represented in the fitness function or a reversion to the wild type at that position were considered by the evolutionary model. Other nucleotide mutations were not considered, as if they were lethal.

(1) **for** k **in** $m_k(N)$:
(1.1) $s_k \leftarrow \frac{F(m_k(N))}{F(N)} - 1$
(1.2) $t_k \leftarrow$ draw from $T_{50}(N_e, s_k, \mu_k)$
(2) $k \leftarrow$ arg min$_k$ (t_k)
(3) $M \leftarrow m_k(N)$

Fig. 2. Algorithm to obtain a sample nucleotide sequence M from the stochastic evolution and fixation of a single nucleotide mutation in the population consenses nucleotide sequence N over a fitness landscape F. $m_k(N)$: every possible nucleotide mutation applied to sequence N; μ_k: the nucleotide mutation rate for mutation k; and N_e: the effective population size of the population.

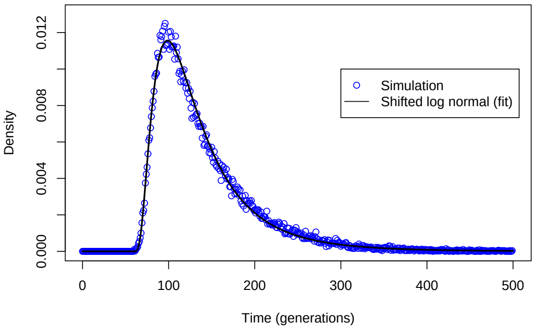

Fig. 3. Example of the density function $T_{50}(N_e, s, \mu)$, time for a mutant with selective advantage $s = 0.01$ and mutation rate $\mu = 10^{-6}$ to reach a prevalence of 50% in a finite population with effective population size $N_e = 10^4$ obtained from simulations. The density function was fit by a shifted log normal distribution with ($a = 59, \mu = 4.1, \sigma^2 = 0.47$).

Intra-host population parameters

For the HIV-1 simulation model, a constant intra-patient effective population size $N_e = 10^4$ was assumed, a value previously estimated from *in vivo* observations during treatment (Nijhuis *et al.*, 1998; Rouzine and Coffin, 1999), and an average mutation rate $\mu = 2.17 \times 10^{-5}$ mutations/site/generation (Mansky and Temin, 1995) was used. Furthermore, we used base-dependent mutation rates $\mu_i = \mu(b_{\text{from}}, b_{\text{to}})$ that were estimated from *in vivo* longitudinal data (Deforche *et al.*, 2007). For the estimation, we used $G_{\max} = 200$, corresponding to about a year of evolution, given an estimated generation turnover time of ± 1.5 days; $L^E = |\mathcal{P}^E| = 10 \times |\mathcal{P}^T|$ and $\epsilon = 10^{-7}$.

Iterative Algorithm for the Estimation of Fitness Function Parameters

The parameters $\phi_{i,k}$ of the function F are estimated so that evolution over the fitness landscape of a naive population $\mathcal{P}^N$ resembles the treated population $\mathcal{P}^T$. Therefore, evolution is simulated for sequences sampled from the naive population $\mathcal{P}^N$ using the fitness function, to obtain an evolved population $\mathcal{P}^E$. The difference between the sequence populations $\mathcal{P}^E$ and $\mathcal{P}^T$, which must thus be minimized, is measured by comparing the parameters of $BN^T(\theta^T, S^T)$ of the treated data set, with $BN^E(\theta^E, S^T)$, a BN estimated from the simulated population using the structure that was learned from the treated data set. Thus, we measure and minimize the difference in prevalence of each mutational pattern that is modeled by the BN, and for which the fitness function specifies a separate fitness contribution.

Fitness function parameters $\phi_{i,k} = F(A_i = 1 \,|\, \text{parents}(A_i) = k)$ were estimated by an iterative algorithm. The algorithm searches for values $\phi_{i,k}$ so that the difference between a population evolved over the landscape, $\mathcal{P}^E$, and the treated population $\mathcal{P}^T$ is minimized.

The algorithm is illustrated with pseudo-code in Figure 4. Starting from a flat fitness landscape, by initializing all $\phi_{i,k}$ to 1 (Figure 4: 1), parameters were adjusted using an iterative procedure. A population $\mathcal{P}^E$ was computed by sampling L^E sequences from the naive population, and evolving them over the current estimate of the fitness landscape (Figure 4: 2.1 – 2.2). To compare this population $\mathcal{P}^E$ with $\mathcal{P}^T$, the Bayesian network structure S^T was trained with data from the evolved population $\mathcal{P}^E$ to obtain $BN^E(\theta^E, S^T)$. In this way, for every pattern k of parents for mutation A_i, each parameter $\theta^T_{i,k}$ (probability of mutation A_i given that pattern in the treated population) has a corresponding parameter $\theta^E_{i,k}$ (probability in the simulated population) and a fitness landscape parameter $\phi_{i,k}$ (fitness contribution for mutation A_i given that pattern). Each fitness landscape parameter $\phi_{i,k}$ was then adjusted using the difference between $\theta^E_{i,k}$ and $\theta^T_{i,k}$ (Figure 4: 2.4.1): an increase of $\phi_{i,k}$ for a too low prevalence of A_i in the simulated population compared to the treated population, and vice-versa. Uncertainty on these parameters was taken into account by using the *sufficient statistics* $SS_{i,k}$ (Myllymäki *et al.*, 2002) instead of $\theta_{i,k}$. Depending on the sign of the difference $d_{i,k}$, $\phi_{i,k}$ was adjusted with a small multiplicative adjustment factor $\delta_{i,k}$ (Figure 4: 2.4.2). The values $\delta_{i,k}$ were dynamically adjusted depending on the convergence of the corresponding $\phi_{i,k}$: when $d_{i,k}$ changed sign compared to the previous iteration, $\delta_{i,k}$ was decreased, while when the sign of $d_{i,k}$ did not change for a number of consecutive iterations, $\delta_{i,k}$ was increased. Convergence was assessed when all $\delta_{i,k}$, which were initialized to a small number ϵ, dropped below that ϵ.

Convergence properties

The presented algorithm estimates fitness contributions so that the joint probability distribution (JPD) of $\mathcal{P}^E$ resembles the JPD of $\mathcal{P}^T$. This resemblance is measured using a fixed Bayesian Network structure, S^T, instead of using a general distance measure to compare the similarity of two JPDs such as the Kullback-Leibler distance. Therefore, the method does not guarantee the convergence of the JPD of $\mathcal{P}^E$ to the JPD of $\mathcal{P}^T$, since it only takes into account interactions that were included in S^T, estimated from $\mathcal{P}^T$, but not

initialization as flat landscape:
(1) **for all** i, k:
(1.1) $\phi_{i,k} \leftarrow 1$

iteratively update parameters $\phi_{i,k}$ of landscape F:
(2) **repeat until** all $\delta_{i,k} < \epsilon$:
(2.1) $\mathcal{P}^E \leftarrow \emptyset$
(2.2) **repeat** L^E times:
(2.2.1) $N \leftarrow$ sample naive nucleotide sequence
(2.2.2) $g \leftarrow$ sample from $P(G^T)$
(2.2.3) $N' \leftarrow$ evolve N up to g generations over F
(2.2.4) $\mathcal{P}^E \leftarrow \mathcal{P}^E \cup \{N'\}$
(2.3) compute $BN^E(\theta^E, S^T)$ from $\mathcal{P}^E$ and given structure S^T
(2.4) **for all** i, k:
(2.4.1) $d_{i,k} \leftarrow SS^T_{i,k}/|\mathcal{P}^T| - SS^E_{i,k}/|\mathcal{P}^E|$
(2.4.2) $\phi_{i,k} \leftarrow \phi_{i,k}(1+\delta_{i,k})^{sign(d_{i,k})}$
(2.4.3) adjust $\delta_{i,k}$

Fig. 4. Algorithm to estimate the parameters in the fitness landscape by simulating evolution of treatment naive sequences over a current estimate F and adjusting the fitness function parameters $\phi_{i,k}$ so that the difference between an evolved population and a treated population is minimized.

interactions that are present in $\mathcal{P}^E$ and missing from S^T. This is intentional, and motivated by the fact that the fitness function only models individual fitness contributions for interactions included in S^T, and thus can only influence the prevalence of corresponding mutational patterns.

A dependency present in $\mathcal{P}^E$ but not in S^T could occur when the Bayesian network for $\mathcal{P}^T$ does not model a dependency between a mutation M and pattern P, which is present in the treatment-naive population $\mathcal{P}^N$, and reflects perhaps an association established by genetic drift. In that case, the fitness function will not model an individual contribution for a mutation M depending on the presence or absence of the pattern P, and the association will therefore in general be maintained through evolution over the fitness function, and appear in $\mathcal{P}^E$. That the algorithm is expected to converge also when a dependency is present in $\mathcal{P}^E$ but not in $\mathcal{P}^T$, is illustrated below.

Consider a situation with two mutations, A and B. Assume that there exists a dependency of A on B in $\mathcal{P}^E$ that is not modeled by S^T.

Because of the dependency, the prevalence of mutation B could be different in presence or absence of the pattern A, both in the naive population $\mathcal{P}^N$ and in the evolved population $\mathcal{P}^E$:

$$P^E(B|A) \neq P^E(B|\bar{A})$$

where $\bar{A}$ indicates absence of mutation A.

The single fitness parameter $F(B)$ will however have an equal effect on both these prevalences, since $F(B) = F(B|A) = F(B|\bar{A})$, and fitness is the only information that directs evolution of the population. Let's consider two different values for $F(B)$:

$$F_1(B) = \delta F_0(B)$$

and a corresponding multiplicative change π in prevalence of $B|A$ and $B|\bar{A}$ after evolution of the landscape for both fitness values:

$$P_1^E(B|A) = \pi P_0^E(B|A)$$

and

$$P_1^E(B|\bar{A}) = \pi P_0^E(B|\bar{A})$$

Then:

$$\begin{aligned} P_1^E(B) &= P_1^E(B|A)P_1^E(A) + P_1^E(B|\bar{A})P_1^E(\bar{A}) \\ &= \pi P_0^E(B|A)P_0^E(A) + \pi P_0^E(B|\bar{A})P_0^E(\bar{A}) \\ &= \pi P_0^E(B) \end{aligned}$$

Thus, ignoring second order effects, $P^E(B)$ will change because of changes in $F(B)$ in the same way as when the additional interaction would not be present in P^E, Q.E.D.

Second order effects create an inherent coupling between all mutations in the model since an increase (decrease) from $F_0(B)$ to $F_1(B)$ may in general result in a slight decrease (increase) in prevalence of any other mutation, including A, since more evolution of B comes in general at the expense of the same amount less evolution for all other mutations in total.

REFERENCES

Deforche, K., *et al.* (2006) Analysis of HIV-1 pol sequences using Bayesian Networks: implications for drug resistance. *Bioinformatics*, **22**, 2975–9.

Deforche, K., *et al.* (2007) Estimating the relative contribution of dNTP pool imbalance and APOBEC3G/3F editing to HIV evolution in vivo. *J. Comput. Biol.*, **14**, 1105–1114.

Deforche, K., *et al.* (2008) Estimation of an in vivo fitness landscape experienced by HIV-1 under drug selective pressure useful for prediction of drug resistance evolution during treatment. *Bioinformatics*, **24**, 34–41.

Ewens, W. J. (1979) *Mathematical population genetics*. Biomathematics, Vol. 9. Berlin, Heidelberg, New York: Springer-Verlag. XII.

Mansky, L. and Temin, H. (1995) Lower in vivo mutation rate of human immunodeficiency virus type 1 than that predicted from the fidelity of purified reverse transcriptase. *J Virol*, **69**, 5087–5094.

Myllymäki, P., *et al.* (2002) B-Course: a web-based tutorial for Bayesian and caausal data analysis. *Int J on Art Intell Tools*, **11**, 396–387.

Nijhuis, M., *et al.* (1998) Stochastic processes strongly influence HIV-1 evolution during suboptimal protease-inhibitor therapy. *PNAS*, **95**, 14441–14446.

Rouzine, I. M. and Coffin, J. M. (1999) Linkage disequilibrium test implies a large effective population number for HIV in vivo. *PNAS*, **96**, 10758–10763.

Wang, Y. and Rannala, B. (2004) A Novel Solution for the Time-Dependent Probability of Gene Fixation or Loss Under Natural Selection. *Genetics*, **168**, 1081–1084.
